# Online behavioural activation during the COVID-19 pandemic decreases depression and negative affective bias

**DOI:** 10.1017/S0033291721002142

**Published:** 2021-08-17

**Authors:** Tereza Ruzickova, James Carson, Stirling Argabright, Amy Gillespie, Calum Guinea, Anna Pearse, Robbie Barwick, Susannah E. Murphy, Catherine J. Harmer

**Affiliations:** 1 University Department of Psychiatry, Warneford Hospital, Oxford, UK; 2 Oxford Health NHS Foundation Trust, Warneford Hospital, Oxford, UK; 3 Lifespan Brain Institute of Children's Hospital of Philadelphia and Penn Medicine, Philadelphia, USA; 4 Medical Sciences Division, University of Oxford, Oxford, UK; 5 Central and North West London NHS Foundation Trust, London, UK

**Keywords:** Behavioural activation, COVID-19, depression, emotional cognition

## Abstract

**Background:**

The COVID-19 pandemic highlighted the need for mental health interventions that can be easily disseminated during a crisis. Behavioural activation (BA) is a cost-effective treatment that can be administered by non-specialists; however, it is unclear whether it is still effective during a time of lockdown and social distancing, when opportunities for positive activity are significantly constrained.

**Methods:**

Between May and October 2020, we randomised 68 UK participants with mild to moderate low mood to either a 4-week online programme of non-specialist administered BA or to a passive control group. Before and after the intervention, we collected self-report data on mood and COVID-related disruption, as well as measuring emotional cognition as an objective marker of risk for depression.

**Results:**

In comparison to the control group, the BA group showed a significant decrease in depression, anxiety and anhedonia after the intervention, as well as an increase in self-reported activation and social support. Benefits persisted at 1-month follow-up. BA also decreased negative affective bias on several measures of the Facial Emotion Recognition Task and early change in bias was associated with later therapeutic gain. Participants rated the intervention as highly acceptable.

**Conclusion:**

This study highlights the benefits of online BA that can be administered by non-specialists after brief training. These findings can help inform the policy response towards the rising incidence of mental health problems during a crisis situation such as a pandemic. They also highlight the use of objective cognitive markers of risk across different treatment modalities.

## Introduction

The COVID-19 pandemic has brought about unprecedented disruption in most areas of society. The incidence of depression and anxiety has markedly increased (Ettman et al., 2020; Winkler et al., 2020) and may further exacerbate the physical health burden (Shevlin et al., 2020). Moreover, contracting COVID-19 has itself been associated with psychiatric sequelae (Taquet, Luciano, Geddes, & Harrison, 2021), which may increase the rate of mental health issues further.

The mental health impact of the pandemic has also been reflected in research on emotional cognition, a possible objective marker of psychiatric vulnerability (Joormann, Talbot, & Gotlib, 2007). Depression is associated with changes in emotional processing (increased negative *v.* positive bias) which are believed to play a role in the risk and maintenance of the disorder (Disner, Beevers, Haigh, & Beck, 2011). Bland et al. (2021) examined emotional cognition between April and May 2020 in a sample with no previous mental health problems and found significantly reduced recognition of happy faces, particularly in those who experienced greater disruption of social contact. This further supports the evidence that psychological vulnerability has increased during COVID-19 and can be detected using objective cognitive markers.

Several cross-sectional studies have investigated what factors might play a role within this context. One UK study found that high levels of mental wellbeing were associated with higher levels of physical activity (Jacob et al., 2020), while a Spanish survey reported that lifestyle factors, such as following a routine, pursuing hobbies and spending time outdoors, were associated with lower levels of depression (Fullana, Hidalgo-Mazzei, Vieta, & Radua, 2020). Time spent focusing on the pandemic has also been identified as a risk factor (Fullana et al., 2020; Huang & Zhao, 2020). However, due to a lack of randomised controlled studies, it is not possible to infer the direction of causality or investigate whether other variables might better explain these effects.

It is crucial to experimentally examine what interventions may be most appropriate during societal crisis periods. It has been argued that the provision of cognitive-behavioural therapy (CBT) should be increased (da Lopes & Jaspal, 2020); however, this intervention is costly and not easily accessible around the world. It has been found that behavioural activation (BA) can be as effective for depression as CBT but more cost-effective, because it can be delivered by junior mental health workers with shorter training (Richards et al., 2016). BA helps patients examine their daily behaviour and assists them in finding the right balance of routine, pleasurable and necessary activities, which may be particularly helpful at a time of significant lifestyle disruption.

However, it is unclear whether BA can still be effective when options for activities are significantly constrained due to social distancing and whether it can be administered remotely in this context. In addition to the effects on subjective wellbeing, it is also unknown whether it can reduce the cognitive markers of vulnerability to depression discussed above. Previous work has suggested that emotional cognitive measures can be sensitive to the therapeutic effects of antidepressant medication prior to effects on mood and may therefore represent an early marker of response (Harmer, Duman, & Cowen, 2017). However, these markers have not been explored in BA for depression and it is unknown whether interventions largely targeting behaviour would also influence these kinds of cognitive processes.

### Current study

In this randomised controlled study, we investigated whether a 4-week course of remote non-specialist administered BA can reduce depression and anxiety and increase activation during a period of social distancing restrictions. As secondary outcomes, we examined participants' emotional cognition, using a battery of tasks sensitive to different treatments for depression. We also monitored anhedonia, automatic negative thoughts, social support and COVID-related lifestyle disruption. The main aims of the study were therefore to test the efficacy of the intervention as well as examining its possible mechanisms.

## Materials and methods

### Participants

Seventy participants were recruited via community advertising. Two participants dropped out after screening and the final sample included 57 women and 11 men. All participants scored between 10 and 28 on the Beck Depression Inventory (BDI-2) at baseline. Exclusion criteria included undergoing any other psychological treatment, reporting suicidal thoughts, having a past or present diagnosis of psychosis or bipolar disorder or having a current diagnosis of an eating disorder, borderline personality disorder, substance abuse, OCD or PTSD (as assessed using the Structured Clinical Interview for DSM-5). Recruitment was carried out between May and October 2020.

### Intervention

We followed an established BA programme ‘Get Active, Feel Good’ (Farrand, Taylor, Greaves, & Pentecost, 2013) routinely used in the UK Improving Access to Psychological Therapies (IAPT) services. Our intervention lasted 4 weeks and it was administered by two psychology researchers who were trained and supervised by a Psychological Wellbeing Practitioner (PWP) from a local IAPT service. Each researcher received 15 h of training prior to the study with special emphasis on adapting the programme for remote administration. Afterwards, each researcher submitted recordings of their practice sessions for evaluation, and once recruitment started, ongoing support from the PWP was available. Participants had an initial introductory session lasting 1.5 h which included psychoeducation, goal setting, baseline activity monitoring and activity planning for the first week, following the official programme booklet. After that, participants had a 30 min meeting each week to discuss which activities they accomplished, to problem solve and to make a plan for the following week (four additional meetings in total). The control group did not receive any intervention but received materials about BA at the end of the study. All meetings were carried out remotely using Microsoft Teams.

### Questionnaire measures

We measured symptoms of depression using the BDI-2, anxiety using the State-Trait Anxiety Inventory (STAI), activation using the Behavioural Activation for Depression Scale (BADS), social support using the Multidimensional Scale of Perceived Social Support (MSPSS), anhedonia using the Snaith–Hamilton Pleasure Scale (SHAPS) and automatic negative thoughts using the Automatic Thoughts Questionnaire (ATQ).

We measured COVID-19-related lifestyle disruption using several questionnaires developed in our department (see online Supplementary materials). The Current Stressors Questionnaire asked about the extent to which participants have been stressed about 18 life domains (including finances, getting medication or job security) over the past week on a four-point Likert scale. The COVID Anxiety Questionnaire asked participants to evaluate nine statements (such as ‘I am worried that I will catch COVID-19’) on a five-point Likert scale.

The Current Disruption Questionnaire asked participants to evaluate how disrupted six different life areas, such as work, friendship or leisure time, had been on a four-point Likert scale. Participants were also asked to report if they were in a high-risk COVID-19 category and whether they had previously tested positive for COVID-19. We also asked participants about several aspects of their living and work situation in relation to the pandemic (see online Supplementary materials).

We sent electronic daily mood questionnaires to participants based on the Mood Zoom format (Tsanas et al., 2016), asking participants to rate the extent to which they feel happy, energetic, anxious, angry, tired and irritable on a scale from 1 to 7. BA participants' experience of the intervention was assessed in a final feedback questionnaire (see online Supplementary materials).

### Cognitive tasks

All tasks were delivered online via the Gorilla platform. In the Facial Emotion Recognition Task (FERT; see Harmer et al., 2003), participants were presented with faces of four actors (two male and two female) showing the six basic emotions (anger, disgust, fear, happiness, sadness and surprise) at different levels of intensity (from 10 to 100%, increasing in 10% steps) alongside neutral faces. Each face was presented for 500 ms on a black screen and participants were asked to identify the emotion as quickly and accurately as possible. A different set of stimuli (same emotions expressed by different actors) was presented in each of the three runs of the task. We measured percentage accuracy and misclassification counts, comparing responses to neutral faces, faces of positive valence (happiness and surprise) and negative valence (anger, disgust, fear and sadness), as well as comparing emotions individually.

In the Emotion Categorisation Task (ECAT; see Chan, Harmer, Goodwin, & Norbury, 2008), participants were presented with 40 randomised personality adjectives (such as ‘cheerful’ or ‘dishonest’), 20 positive and 20 negative, each presented for 500 ms at a time. They were asked to indicate whether they would like or dislike to be described as the word by pressing a ‘like’ or ‘dislike’ button as quickly and accurately as possible. We measured accuracy and reaction times. Different sets of words were presented at each of the three runs. In the Emotional Recall Task (EREC; see Harmer, Shelley, Cowen, & Goodwin, 2004), which took place 15 min later, participants were asked to remember as many words as possible from the ECAT task. We measured the accuracy of positive and negative recall, as well as the number of positive and negative intrusions.

In the Probabilistic Instrumental Learning Task (PILT; see Walsh, Browning, Drevets, Furey, & Harmer, 2018), participants started with 100 pence and were told to try to win as much money as possible by picking between different abstract symbols, with the final amount won being added to their reimbursement. On each trial, they were shown two symbols which had reciprocal probabilities (0.7 *v.* 0.3) of either winning *v.* not winning money (in ‘gain’ trials) or not losing *v.* losing money (in ‘loss’ trials). After the choice, participants were given feedback about their monetary outcome (20 pence won, 20 pence lost or no change) and their current total. A different set of stimuli was used at each run of the task. The main outcome measures were total amount won and lost, percentage accuracy for choosing high probability symbols across the whole task and in the last 20 trials.

### Procedure

Eligible participants were randomly allocated to either the BA intervention or to a control group, stratified by sex. Questionnaire measures (BDI-2, BADS, STAI, SHAPS, ATQ, MSPSS as well as all COVID-related questionnaires) were administered remotely at baseline (week 0), halfway through the intervention (week 2) and at the end (week 4) using the Qualtrics software. A link to the daily Mood Zoom questionnaire was texted to participants every evening using the Text Marketer platform.

Cognitive tasks (FERT, ECAT, EREC and PILT) were administered remotely at baseline (week 0), halfway through the intervention (week 2) and at the end (week 4). Multiple choice questions designed to test for understanding of the task instructions had to be completed before participants could proceed with each task.

One month after finishing the study, all participants were asked to fill in a follow-up assessment, which included the BDI-2, STAI, BADS and all COVID-related measures.

### Statistical analysis

Questionnaire measures were analysed using two-way mixed ANOVAs with group as a between-subject factor (BA and control) and time as a within-subject factor (baseline, week 2, week 4 and follow-up at week 8 where relevant). Measures of emotional cognition were analysed as change-from-baseline scores (week 4–0 and week 2–0) using a two-way ANCOVA controlling for baseline, with group as a between-subject factor and time (week 2 *v.* 4) and valence (positive or negative) or emotion (anger, disgust, fear, happiness, sadness, surprise or neutral) as within-subject factors. Change-from-baseline scores were further correlated between emotional cognitive measures at week 2 and depression scores at week 4 and 8 while controlling for baseline. Mood Zoom data were analysed with three groups of valences: positive (happy, energetic), negative (sad, anxious) and irritable (irritable, angry). The PILT had two groups of trial type (win *v.* loss) instead of valence as a within-subject factor.

Significant interactions were followed up with simple main effect analyses; independent *t* tests were used for simple main effects of group and pairwise comparisons, separated by group, were used for simple main effects of time.

Normality was assessed using the Shapiro–Wilk test. Outliers were assessed by the inspection of box plots and studentised residuals; they were removed if they were 3 box-lengths away from the edge of the box or if the studentised residual was greater than ±3 standard deviations. Mauchly's test was used to assess the assumption of sphericity; if sphericity was violated, Greenhouse–Geisser correction was applied. Box's test was used to assess the equality of covariance.

All data were processed and analysed using R and SPSS software. For a sensitivity analysis, all data were compared with and without the 13 participants who were taking antidepressants to investigate whether any effects may have been driven by the concurrent pharmacological treatment.

## Results

### Demographic, clinical and COVID-related baseline information

The two groups were well matched in terms of the main demographic and clinical characteristics at baseline (see Table 1).

**Table 1.** Demographic, clinical and COVID-related baseline characteristics (for further variables see online Supplementary materials)

Variable	BA group (*n* = 34)	Control group (*n* = 34)
Age	32.38 (10.92)	30.79 (11.27)
Years in full time education	16.29 (3.23)	15.88 (2.29)
Race	76.5% white,	96.9% white,
	23.5% non-white	3.1% non-white
Current antidepressant treatment	14.7%	23.5%
Baseline work status	25.7% full time	37% full time
	25.7% part time	20% part time
	48.6% unable to work	43% unable to work
Baseline COVID-19 risk	100% no	94% no
		6% yes
Baseline COVID-19 symptoms	100% no	97% no
		3% yes
Baseline COVID-19 diagnosis	97% no	80% no
	3% suspected	20% suspected

### Rating of intervention

Feedback forms were returned by 91% of BA participants and the intervention was rated as acceptable, with a mean 82% rating of the intervention as helpful and a 92% rating for stating that they would recommend it to others during lockdown (for all ratings, see online Supplementary materials).

### Effects on self-report measures

**Fig. 1.**
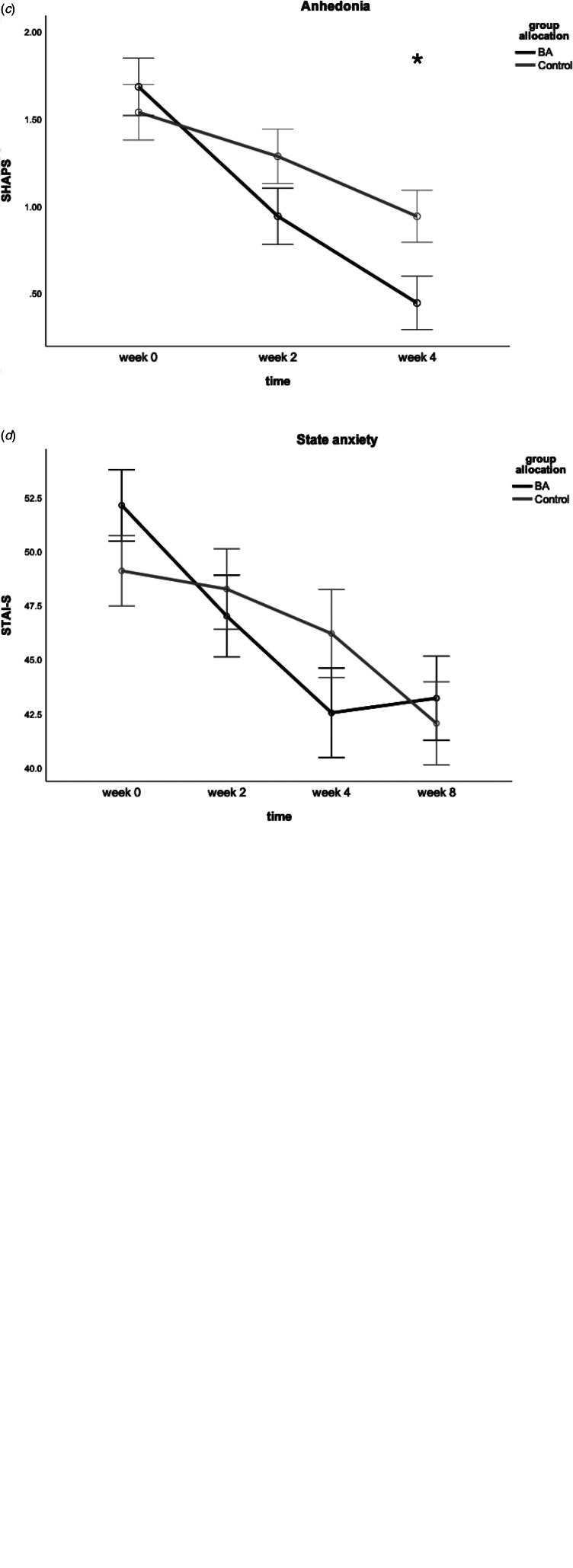
Plots showing mean scores in main self-report measures for behavioural activation (BA) group and control group over the 4-week intervention and at 1-month follow-up where relevant. (*a*) Depression scores as measured by Beck Depression Inventory 2, (*b*) behavioural activation scores measured by Behavioural Activation for Depression Scale, (*c*) anhedonia as measured by Snaith–Hamilton Pleasure Scale (after square transformation), (*d*) state anxiety as measured by State-Trait Anxiety Inventory. Asterisk indicates *p* < 0.05. Error bars show ±1 standard error.

There was a significant time by group interaction for depression scores [*F*_(3,189)_ = 7.75, *p* < 0.001, *η*^2^ = 0.11]. Follow-up analyses revealed that this interaction was driven by a significant difference between groups at the end of the intervention [*t*(65) = −2.68, *p* = 0.009, *d* = 0.66], with the BA group showing lower depression scores than the control group, see Fig. 1*a*.

The significant difference persisted at 1-month follow-up [*t*(65) = −3.00, *p* = 0.004, *d* = 0.74], again with depression symptoms significantly lower in the BA group.

There was also a significant time × group interaction for self-reported activation scores, as measured by the BADS [*F*_(3,183)_ = 4.75, *p* = 0.003, *η*^2^ = 0.07]. Follow-up analyses revealed significant differences between the groups midway through the intervention at week 2 [*t*(64) = 2.12, *p* = 0.04, *d* = 0.52], at the end of intervention at week 4 [*t*(65) = 2.99, *p* = 0.004, *d* = 0.73] and at 1-month follow-up [*t*(65) = 2.16, *p* = 0.04, *d* = 0.53] with the BA group showing higher activation, see Fig. 1*b*.

Our data on anhedonia (shown in Fig. 1*c*) were moderately positively skewed and square root transformation was applied to prevent violations of ANOVA assumptions. The measure showed a significant time × group interaction [*F*_(1.61,102.75)_ = 4.07, *p* = 0.03, *η*^2^ = 0.06], which was driven by a significant difference between groups at final time point [*t*(64) = −2.32, *p* = 0.02, *d* = 0.58].

There was also a significant time × group interaction for state anxiety [*F*_(3,183)_ = 2.75, *p* = 0.04, *η*^2^ = 0.04], see Fig. 1*d*. Simple main effects of group did not show any differences between groups at any time point (*p* > 0.05). Simple main effects of time showed that the BA group showed significant differences between baseline and midway [*t*(31) = 2.93, *p* = 0.006, *d* = 0.48], baseline and end of intervention [*t*(31) = 4.10, *p* < 0.001, *d* = 0.83] and baseline and follow-up [*t*(31) = 4.10, *p* < 0.001, *d* = 0.78]. The control group only showed a significant difference between baseline and follow-up [*t*(32) = 3.82, *p* = 0.001, *d* = 0.66]. For trait anxiety, there was only a trend towards a significant time × group interaction (*p* = 0.07).

Levels of social support were measured by the MSPSS and showed significant time × group interaction [*F*_(2,120)_ = 5.21, *p* = 0.007, *η*^2^ = 0.08]. Simple main effects of group did not show any differences between groups at any time point (*p* > 0.05). Simple main effect of time analysis showed that the BA group had a significant increase in social support scores between baseline (*M* = 59.09, s.d. = 12.79) and midway [*M* = 62.34, s.d. = 11.59; *t*(31) = −2.47, *p* = 0.02, *d* = 0.27] and baseline and end of intervention [*M* = 62.52, s.d. = 11.80; *t*(30) = −2.75, *p* = 0.01, *d* = 0.28]. The control group did not show any significant differences between any time points (*p* > 0.05).

Automatic negative thoughts, as measured by the ATQ, showed no significant time × group interaction [*F*_(2,122)_ = 0.42, *p* = 0.66, *η*^2^ = 0.007] but showed a significant main effect of time [*F*_(2,122)_ = 15.46, *p* < 0.001, *η*^2^ = 0.20]. This was driven by a significant decrease in negative thoughts from baseline (*M* = 34.97, s.d. = 9.77) to midway time point (*M* = 32.33, s.d. = 10.43, *p* = 0.02), as well as from baseline to final time point (*M* = 29.05, s.d. = 10.43, *p* < 0.001) for all participants.

Due to a large amount of missing data in week 4 of the Mood Zoom dataset, we only analysed the first three weeks of intervention. There was no time × group × valence interaction [*F*_(4,260)_ = 1.25, *p* = 0.29, *η*^2^ = 0.02].

There was a significant main effect of time for COVID-related stress [*F*_(2.48,160.93)_ = 3.25, *p* = 0.03, *η*^2^ = 0.05], COVID-related lifestyle disruption [*F*_(2.36,153.67)_ = 33.37, *p* < 0.001, *η*^2^ = 0.34] and COVID-related anxiety [*F*_(2.09,131.37)_ = 11.75, *p* < 0.001, *η*^2^ = 0.16], which reflected a decrease in these measures across the study in both groups.

### Sensitivity analysis: effect of antidepressant treatment

Exclusion of participants on antidepressants did not affect the interaction effects for any of the questionnaire measures.

### Effects on emotional cognition

#### Facial Emotion Recognition Task (FERT)

**Fig. 2.**
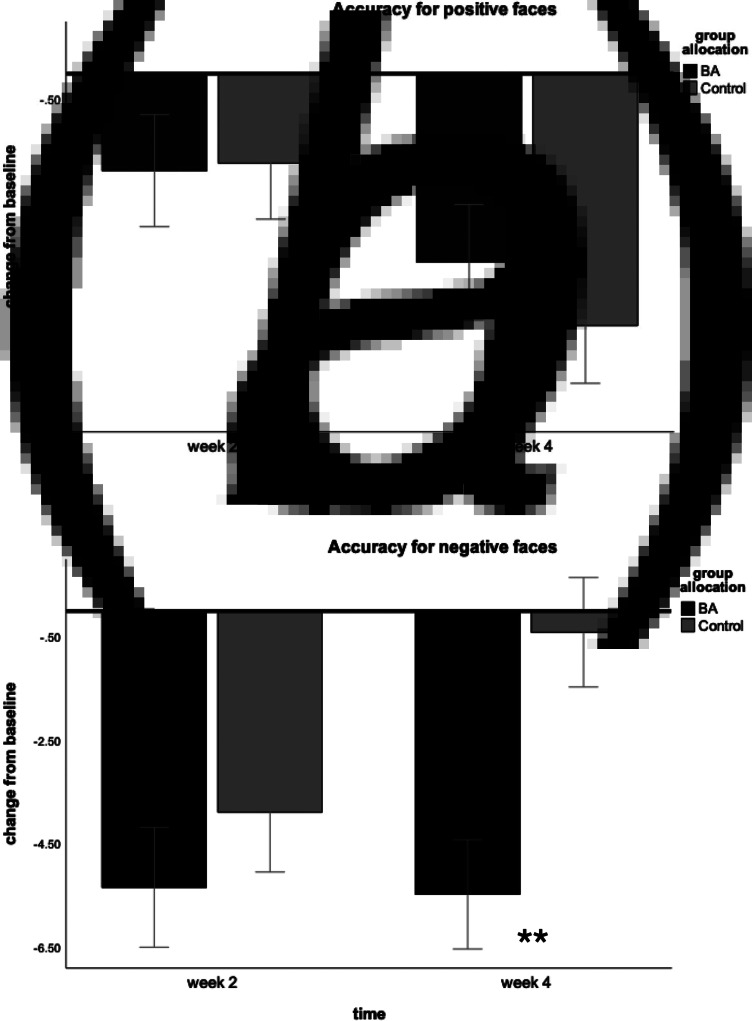
Bars showing mean change from baseline in accuracy towards identifying positive and negative emotions at week 2 and 4 in the Facial Emotion Recognition Task (controlling for baseline). Week 2 represents midway point in the intervention and week 4 represents end of intervention. Two asterisks indicate *p* = 0.001. Error bars show ±1 standard error.

FERT accuracy showed a significant time × group × valence interaction [*F*_(1,60)_ = 4.36, *p* = 0.04, *η*^2^ = 0.07]. There was no significant difference in accuracy for positive faces at week 2 or 4 (*p* > 0.05), see Fig. 2*a*. The interaction was driven by a significant difference in accuracy for negative faces at week 4 [*t*(63) = −3.36, *p* = 0.001, *d* = 0.83]; with the BA group decreasing in negative accuracy significantly more, see Fig. 2*b*.

Preliminary post-hoc analysis of individual emotions showed that the control group had a significantly higher accuracy for identifying fear at week 4 (see online Supplementary material).

**Fig. 3.**
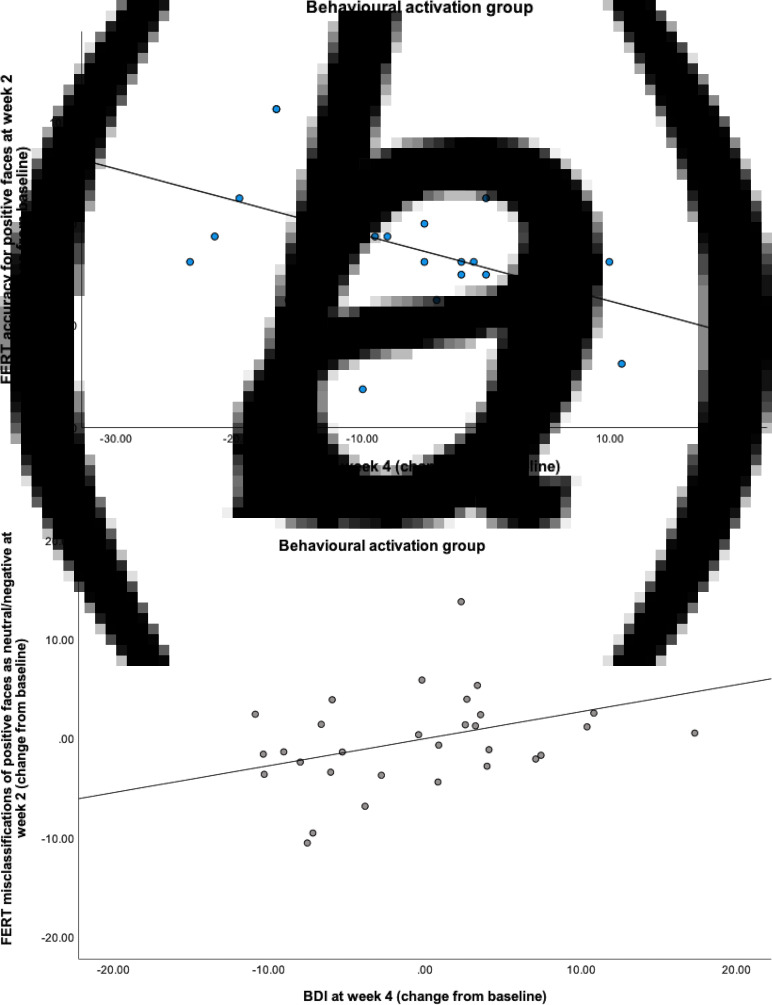
Facial Emotion Recognition Task results of the BA group only. (*a*) A significant negative correlation between a change in accuracy for recognising positive emotions at week 2 and change in depression scores on the Beck Depression Inventory (BDI-2) at week 4. (*b*) A significant positive correlation between a change in misclassifications of positive faces as negative or neutral (negative bias) at week 2 and change in depression scores at week 4. These results suggest that early changes in facial recognition were associated with later therapeutic gain.

Only the BA group showed a significant negative correlation between change in accuracy for positive faces at week 2 and depression scores at week 4 [*r*(27) = −0.41, *p* = 0.03], see Fig. 3*a*. Moreover, only the BA group showed a significant positive correlation between misclassifications of positive faces as negative or neutral (negative bias) at week 2 and depression scores at week 4 [*r*(27) = 0.41, *p* = 0.03], see Fig. 3*b*. This suggested that early change in the processing of positive faces was associated with later therapeutic gain following treatment.

**Fig. 4.**
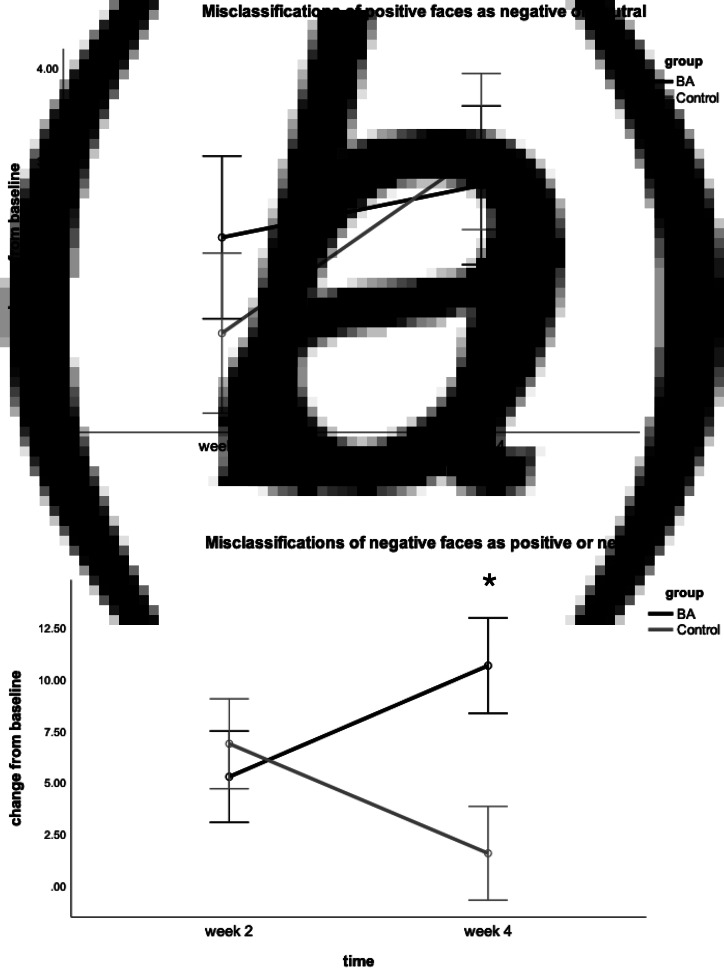
Change from baseline in the mean number of misclassifications in the Facial Emotion Recognition Task. Week 2 represents midway point in the intervention and week 4 represents end of intervention. Figure (*a*) shows misclassifications of positive faces as negative or neutral (negative bias) and (*b*) shows misclassifications of negative faces as positive or neutral (positive bias). The asterisk indicates *p* < 0.05. Error bars show ±1 standard error.

When analysing the number of misclassifications of positive faces as either negative or neutral, there was no significant time × group interaction [*F*_(1,61)_ = 1.15, *p* = 0.30, *η*^2^ = 0.02], see Fig. 4*a*.

For misclassifications of negative faces as positive or neutral, there was a significant time × group interaction [*F*_(1,61)_ = 6.65, *p* = 0.01, *η*^2^ = 0.10]. The interaction was driven by a significant difference between groups at the final time point, wherein the BA group showed a significantly greater increase in misclassifications than the control group [*t*(64) = 2.52, *p* = 0.01, *d* = 0.62], see Fig. 4*b*. There was no significant time × group interaction for misclassifying negative faces as other negative emotions [*F*_(1,61)_ = 0.39, *p* = 0.54, *η*^2^ = 0.006].

When comparing significant differences in individual emotions (see online Supplementary materials), the BA group was found to misclassify fear as other emotions while the control group misclassified surprise. When examining which emotions were falsely selected overall, the BA group selected neutral expression significantly more, while the control group selected fear.

#### Emotional categorisation task (ECAT)

There were no differences in this task between groups [accuracy: *F*_(1,54)_ = 1.77, *p* = 0.19, *η*^2^ = 0.03; reaction time: *F*_(1,52)_ = 1.26, *p* = 0.27, *η*^2^ = 0.02].

#### Emotional Recall Task (EREC)

There was no effect of treatment for either accurate emotional recall [*F*_(1,61)_ = 0.04, *p* = 0.83, *η*^2^ = 0.001] or intrusions [*F*_(1,61)_ = 1.92, *p* = 0.17, *η*^2^ = 0.03]. However, only the BA group showed a significant positive correlation between the change in negative intrusions at week 2 and the change in depression at week 4 [*r*(27) = 0.38, *p* = 0.04], suggesting that reduction in negative intrusions may be related to later therapeutic gain.

#### Probabilistic Instrumental Learning Task (PILT)

There was no effect of treatment in terms of choice behaviour [full task *F*_(1,60)_ = 0.10, *p* = 0.76, *η*^2^ = 0.002; last 20 trials *F*_(1,63)_ = 0.01, *p* = 0.94, *η*^2^ < 0.001]. There was also no time × valence × group interaction for the total amount won and lost [*F*_(1,65)_ = 1.57, *p* = 0.21, *η^2^* = 0.02].

#### Sensitivity analysis: effect of antidepressant treatment

After the exclusion of participants on antidepressants, the interaction effects for FERT accuracy and FERT misclassifications were no longer significant. All other results remained the same.

## Discussion

This study investigated whether non-specialist, online BA can be effective for treating mild to moderate low mood during the COVID-19 pandemic, when options for activities are significantly constrained, and whether this can be detected objectively as a change in affective bias. We found that the intervention had a significant positive effect on our primary measures with medium to large effect sizes, decreasing participants' ratings of depression and state anxiety and increasing activation. The intervention also led to a more positive affective bias, detected as a significant decrease in accuracy for recognising negative faces and their increased misclassification as positive or neutral, again with medium to large effect sizes.

Our study adds further evidence as to the flexibility with which BA can be administered online, making it safe during a period of social distancing. Since it was administered by two researchers just after 15 h of training and under the supervision of a PWP, this suggests that new non-specialist practitioners could be trained very quickly during a period of heightened mental health risk in the population. The intervention was rated as acceptable by the participants and it also increased their rating of social support and decreased anhedonia. This demonstrates that BA may be a particularly helpful intervention during a public health crisis, as it is more cost-effective than full CBT (Richards et al., 2016).

To our knowledge, this is the first study to find evidence of a purely behavioural treatment for depression affecting negative cognitive bias in facial expression recognition. This bias has been associated with depression and vulnerability to depression (Disner et al., 2011), possibly because it makes negative cognitive schemas more likely to be reinforced. It is therefore notable that BA shifted the identification of negative faces towards more positive or neutral interpretations. This provides evidence for the theoretical model of BA (Farrand et al., 2013), wherein bidirectional effects occur between cognition and behaviour (as well as physical sensations).

Consistent with this, BA participants who showed early cognitive changes in three of our cognitive measures at week 2 were more likely to show clinical improvement at week 4. This suggests a possible mechanistic role of affective cognition, wherein positive shifts in unconscious bias may accumulate to eventually shift conscious mood. Such effects have previously been shown mainly for pharmacological treatment for depression (see Harmer et al., 2017 for review), and while some evidence from psychological treatment exists, it has been inconsistent (Porter et al., 2016; Yılmaz et al., 2019). This work highlights how emotional cognition may serve as an early marker of response across different interventions, which may be more resistant to the placebo and demand effects that occur in self-report.

While we saw significant effects of the intervention on our primary outcome measures of depression, activation and facial emotion recognition, several of our secondary measures improved for all participants across time. Across both groups, we found significant decreases in automatic negative thoughts as well as COVID-related stress, anxiety and disruption. This indicates a degree of spontaneous recovery, possibly as participants adapted to the circumstances of the pandemic. Considering the period of recruitment (May–October 2020), most participants experienced the UK lockdown measures as either stable or gradually improving, rather than becoming stricter, which may have impacted these measures.

One possible limitation of our study was the high proportion of females in our sample. Research has shown that women may be particularly at risk of depression during COVID-19 (Daly, Sutin, & Robinson, 2020), which may explain their greater interest in our study. An additional limitation is the lack of control we had over the conditions under which participants completed online data collection. We aimed to minimise disruption through engagement checks and by contacting each participant before each assessment to remind them of task instructions.

## Conclusion

Our study presents compelling evidence that online BA can be a helpful intervention to disseminate during a public health crisis when the need for effective mental health treatments increases. Crucially, non-specialists may be trained quickly to supplement oversubscribed mental health services. We also provide the first evidence that behavioural interventions can affect emotional cognition, providing a way to test their efficacy objectively and highlighting a potential mechanism by which their clinical effects occur.

## Supplementary material

For supplementary material accompanying this paper visit http://dx.doi.org/10.1017/S0033291721002142. click here to view supplementary material
