# Supplementary material for: Online behavioural activation during the COVID-19 pandemic decreases depression and negative affective bias
Source: Psychol Med. 2021 Aug 17:1–10. doi: 10.1017/S0033291721002142 (PMC8438352; doi:10.1017/S0033291721002142)
Supplement: Supplementary file 1 [file S0033291721002142sup001.docx]

Supplementary material for the submission of “Online behavioural activation during the COVID-19 pandemic decreases depression and negative affective bias”

Tereza Ruzickova^1^, James Carson^1^, Stirling Argabright^2^, Amy Gillespie^1^, Calum Guinea^1^, Anna Pearse^3^, Robbie Barwick^4^, Susannah Murphy^1*^, Catherine J Harmer^1*^

^1^ University Department of Psychiatry, Warneford Hospital, Oxford, UK; Oxford Health NHS Foundation Trust, Warneford Hospital, Oxford, UK

^2^ Lifespan Brain Institute of Children’s Hospital of Philadelphia and Penn Medicine, Philadelphia, US

^3^ Medical Sciences Division, University of Oxford, Oxford, UK

^4^ Central and North West London NHS Foundation Trust, London, UK

^*^ *Joint senior author*

**Corresponding author:**

Prof. Catherine Harmer

Psychopharmacology and Emotion Research Laboratory (PERL)

Department of Psychiatry

Warneford Hospital

OX3 7JX

University of Oxford

United Kingdom

e-mail: [catherine.harmer@psych.ox.ac.uk](mailto:catherine.harmer@psych.ox.ac.uk)

**COVID lifestyle questionnaires**

1. **COVID Anxiety Questionnaire**

The following questions will ask you about how you are currently feeling during the COVID-19 outbreak. Please answer the extent to which you agree with each statement.

I think COVID-19 is a very serious issue.

Strongly disagree / Disagree / Neither disagree not agree / Agree / Strongly agree

I am worried that I will catch COVID-19.

Strongly disagree / Disagree / Neither disagree not agree / Agree / Strongly agree

I am worried that friends and family will catch COVID-19.

Strongly disagree / Disagree / Neither disagree not agree / Agree / Strongly agree

I am afraid to leave the house right now.

Strongly disagree / Disagree / Neither disagree not agree / Agree / Strongly agree

I am worried that I might transmit the infection to someone else.

Strongly disagree / Disagree / Neither disagree not agree / Agree / Strongly agree

I am worried we won’t have enough food and other essential items during the outbreak.

Strongly disagree / Disagree / Neither disagree not agree / Agree / Strongly agree

I am worried about missing work.

Strongly disagree / Disagree / Neither disagree not agree / Agree / Strongly agree

I am worried about the amount of money that we have coming in.

Strongly disagree / Disagree / Neither disagree not agree / Agree / Strongly agree

I am worried about the long-term impact this will have on my job prospects and the economy.

Strongly disagree / Disagree / Neither disagree not agree / Agree / Strongly agree

1. **Current Stressors Questionnaire**

Over the past week, how stressed have you felt about the following?

Marriage or other romantic relationship

Not at all / A little / Quite a lot / A great deal / Not applicable

Friends of family living in your household

Not at all / A little / Quite a lot / A great deal / Not applicable

Friends or family living outside your household

Not at all / A little / Quite a lot / A great deal / Not applicable

My child(ren)’s wellbeing

Not at all / A little / Quite a lot / A great deal / Not applicable

Providing home schooling for my child(ren)

Not at all / A little / Quite a lot / A great deal / Not applicable

Household chores

Not at all / A little / Quite a lot / A great deal / Not applicable

Neighbours

Not at all / A little / Quite a lot / A great deal / Not applicable

Loss of usual support systems

Not at all / A little / Quite a lot / A great deal / Not applicable

Living conditions

Not at all / A little / Quite a lot / A great deal / Not applicable

Work (even if you feel your job is safe)

Not at all / A little / Quite a lot / A great deal / Not applicable

Please select the “not at all” option (engagement check)

Not at all / A little / Quite a lot / A great deal / Not applicable

Losing your job/unemployment

Not at all / A little / Quite a lot / A great deal / Not applicable

Finances

Not at all / A little / Quite a lot / A great deal / Not applicable

Getting medication

Not at all / A little / Quite a lot / A great deal / Not applicable

Getting food

Not at all / A little / Quite a lot / A great deal / Not applicable

My own safety/security

Not at all / A little / Quite a lot / A great deal / Not applicable

Internet access

Not at all / A little / Quite a lot / A great deal / Not applicable

Boredom

Not at all / A little / Quite a lot / A great deal / Not applicable

My future plans

Not at all / A little / Quite a lot / A great deal / Not applicable

1. **Current Disruption Questionnaire**

In the past week, how disrupted have you found the following areas of your life?

Work life

Not at all / A little / Quite a lot / A great deal / Not applicable

Friendships

Not at all / A little / Quite a lot / A great deal / Not applicable

Family life

Not at all / A little / Quite a lot / A great deal / Not applicable

Romantic relationships

Not at all / A little / Quite a lot / A great deal / Not applicable

Leisure time

Not at all / A little / Quite a lot / A great deal / Not applicable

Exercise

Not at all / A little / Quite a lot / A great deal / Not applicable

**All demographic, clinical and COVID-related baseline characteristics**

Diagnostic categories were assessed using the Structured Clinical Interview for DSM-5. MDE = major depressive episodes, PDD = persistent depressive disorder, GAD = generalised anxiety disorder

| **Variable (mean, SD)** | **BA group (n = 34)** | **Control group (n = 34)** |
| --- | --- | --- |
| Age | 32.38 (10.92) | 30.79 (11.27) |
| Years in full time education | 16.29 (3.23) | 15.88 (2.29) |
| Race | 76.5% white,  23.5% non-white | 96.9% white,  3.1% non-white |
| Highest education level attained | 26.5% A-level/GCSE  38.2% Undergraduate or professional qualification  35.3% Postgraduate | 23.5% A-level/GCSE  50% Undergraduate or professional qualification  26.5% Postgraduate |
| Current antidepressant treatment | 14.7% | 23.5% |
| Current MDE | 48.5% | 41.2% |
| Current PDD | 6.1% | 5.9% |
| Current GAD | 6.1% | 8.8% |
| Current panic disorder | 6.1% | 2.9% |
| Current social anxiety disorder | 0% | 2.9% |
| Baseline work status | 25.7% full time  25.7% part time  48.6% unable to work | 37% full time  20% part time  43% unable to work |
| Percentage critical key workers as defined by the UK government^[[1]](#footnote-1)^ | 14% yes  86% no | 17% yes  83% no |
| Baseline isolation status | 44.1% only essentials  41.2% social distancing  8.8% normal  5.9% shielding with access to outside space  0% shielding with no access to outside space | 41.2% only essentials  52.9% social distancing  2.9% normal  0% shielding with access to outside space  2.9% shielding with no access to outside space |
| People in household (mean, SD) | 2.79 (1.18) | 2.44 (1.12) |
| Rooms in household (mean, SD) | 5.00 (2.62) | 4.59 (2.12) |
| Outdoor access | 88.2% yes  11.8 % no | 85.3% yes  14.7% no |
| Baseline time spent exercising per week (self-report) | 51% less than 30min  49 % more than 30min | 77% less than 30min  23% more than 30min |
| Baseline time spent outside per week (self-report) | 80% less than 2h  20% more than 2h | 77% less than 2h  23% more than 2h |
| Baseline COVID-19 risk | 100% no | 94% no  6% yes |
| Baseline COVID-19 risk in the family | 77% no  23% yes | 80% no  20% yes |
| Baseline COVID-19 symptoms | 100% no | 97% no  3% yes |
| Baseline COVID-19 diagnosis | 97% no  3% suspected | 80% no  20% suspected |
| Baseline COVID-related stress (score out of 72) | 32.40 (6.44) | 31.57 (7.86) |
| Baseline COVID-related anxiety  (score out of 45) | 32.31 (4.36) | 30.63 (4.66) |
| Baseline COVID-related lifestyle disruption (score out of 24) | 17.40 (3.35) | 16.91 (3.82) |

**Participants' self-reported rating of the intervention (mean rating on 0-100 scale)**

|  | Mean, SD |
| --- | --- |
| Overall, did you find the intervention helpful? | 81.58 (17.61) |
| Did the intervention match your ideas of what helps people with low mood? | 72.97 (21.29) |
| Was the intervention well explained? | 95.97 (6.90) |
| Did you get on well with your BA practitioner? | 97.35 (6.14) |
| Was your BA practitioner supportive and empathetic? | 97.68 (5.49) |
| Do you think you'll continue using the strategies learnt in this intervention? | 86.39 (19.77) |
| Would you recommend this intervention to other people experiencing low mood during the lockdown? | 92.23 (12.75) |

**FERT analysis of individual emotions**

When comparing accuracy to individual emotions, there was a significant three way interaction (*F*(6,324) = 2.60, *p* = .02, *η^2^* = .05), but the subsequent group x emotion interactions at different time points were not significant (*p* > .05). As shown in figure 5, simple main effect of group analysis showed that the control group had a significantly higher accuracy when recognizing fear at the end of the intervention (*t*(64) = -2.07, *p* = .04, *d* = .81).


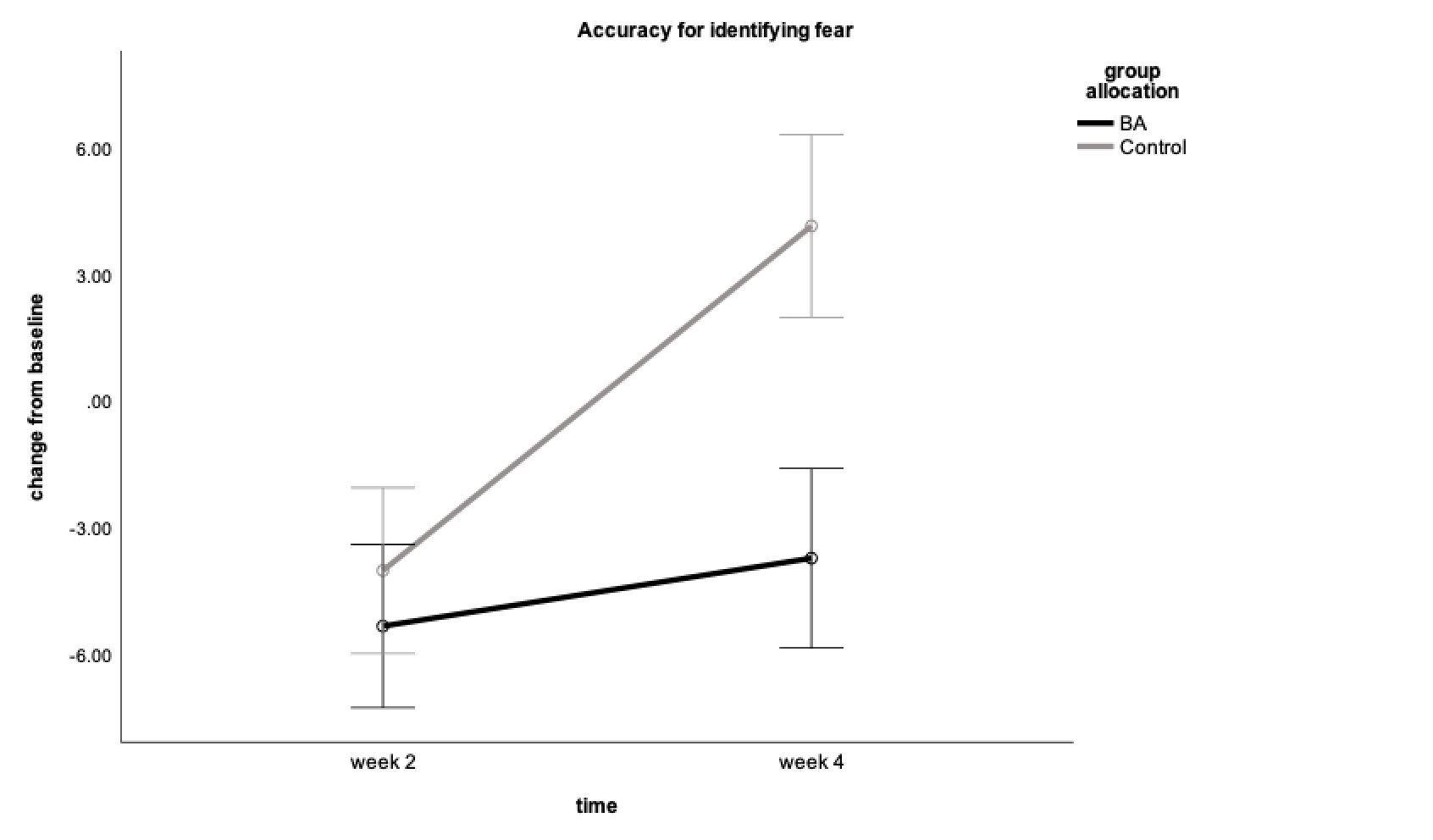


**Figure 5.** Analysis of individual emotion accuracy in the Facial Emotion Recognition Task showed that the control group was significantly better at recognizing fear at the end of the intervention. Error bars show ± 1 standard error.

When comparing how individual emotions were misclassified, there was a significant three way interaction (*F*(6,306) = 3.69, *p* = .001, *η^2^* = .07). A significant group by emotion interaction persisted only at the final time point (F(6,324) = 2.86, p = .01, *η^2^* = .05).

As shown in Figure 6, this was driven by a significant difference between groups in how fear was misclassified (*t*(63) = 2.42, *p* = .02, *d* = .60), with the BA group being more likely to misclassify fear as other emotions and the control group more likely to misclassify surprise as other emotions (*t*(64) = -2.22, *p* = .03, *d* = .55).


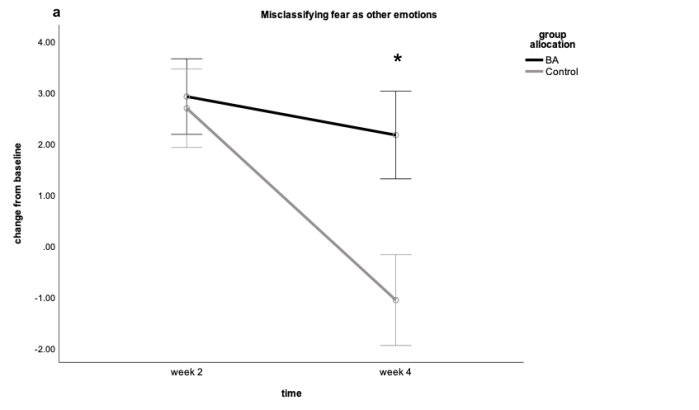

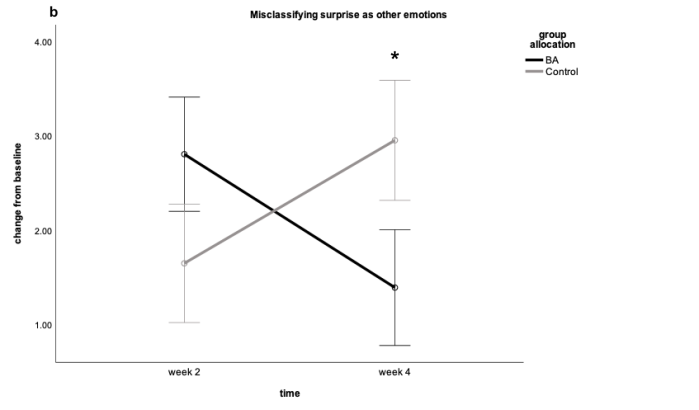


**Figure 6.** Rates of misclassification in the Facial Emotion Recognition Task. a) the BA group misclassifying fear as

other emotions and b) the control group misclassifying surprise significantly more at the end of the intervention. Error bars show ± 1 standard error.

When comparing what emotions were most likely to be falsely selected, there was again a significant three way interaction (*F*(6,270) = 3.90, *p* = .001, *η^2^* = .08) and a significant group by emotion interaction persisted only at the final time point (*F*(6,294) = 4.10, *p* = .001, *η^2^* = .08).

As shown in figure 7, this was driven by the control group selecting fear significantly more (*t*(64) = -2.59, *p* = .01, *d* = .64) and the BA group being much more likely to misclassify emotions as neutral (*t*(58.51) = 2.67, *p* = .01, *d* = .66).

**
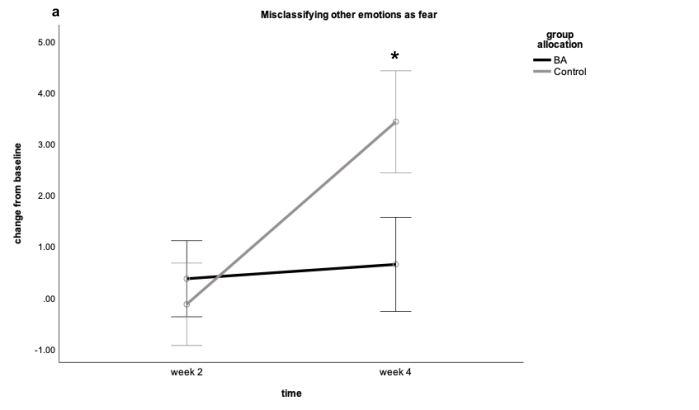

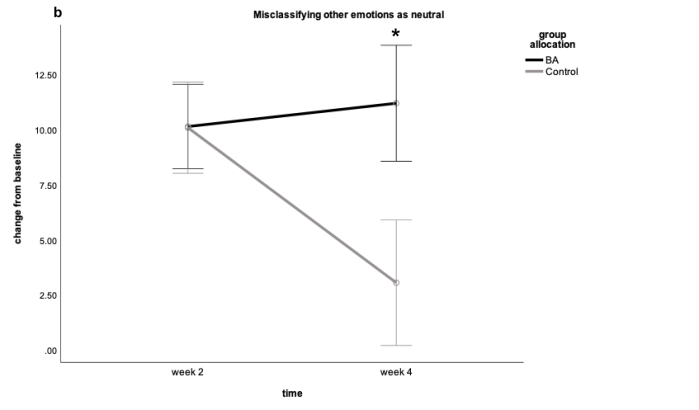
**

**Figure 7.** Falsely selected emotions in the Facial Emotion Recognition Task. a) the control group falsely selecting fear and b) the BA group falsely selecting neutral significantly more at the end of the intervention. Error bars show ± 1 standard error.

1. As defined by Department of Education (2020). *Children of critical workers and vulnerable children who can access schools or educational settings*. (n.d.). GOV.UK. Retrieved 25 March 2021, from [https://www.gov.uk/government/publications/coronavirus-COVID-19-maintaining-educational-provision/guidance-for-schools-colleges-and-local-authorities-on-maintaining-educational-provision](https://www.gov.uk/government/publications/coronavirus-covid-19-maintaining-educational-provision/guidance-for-schools-colleges-and-local-authorities-on-maintaining-educational-provision) [↑](#footnote-ref-1)
